# Minor contribution of mutations at *iniA *codon 501 and *embC-embA *intergenic region in ethambutol-resistant clinical *Mycobacterium tuberculosis *isolates in Kuwait

**DOI:** 10.1186/1476-0711-8-2

**Published:** 2009-01-15

**Authors:** Al-Anoud Jaber, Suhail Ahmad, Eiman Mokaddas

**Affiliations:** 1 Department of Microbiology, Faculty of Medicine, Kuwait University, Kuwait City, Kuwait

## Abstract

**Background:**

Ethambutol (EMB) is a first-line drug for the treatment of tuberculosis (TB). Resistance to EMB in *Mycobacterium tuberculosis *isolates is mediated by mutations in several genes involved in arabinan synthesis notably three *emb *(arabinosyl transferase) and *iniA *(isoniazid-inducible) genes. Most epidemiologically unrelated EMB-resistant *M. tuberculosis *strains contain mutations at *embB *codons 306, 406 and 497, *embC-embA *intergenic region (IGR) and *iniA *codon 501 (*iniA501*).

**Objective:**

To develop a more comprehensive molecular screen for EMB-resistance detectioamong epidemiologically unrelated EMB-resistant *M. tuberculosis *strains previously analyzed for *embB *codon 306, 406 and 497 mutations by including analysis of mutations at *iniA501 *and in *embC-embA *IGR.

**Methods:**

Fifty consecutive and phenotypically documented EMB-resistant and 25 pansusceptible *M. tuberculosis *strains isolated from 75 different TB patients over a four-year period in Kuwait were analyzed. Mutations at *iniA501 *were detected by PCR amplification followed by restriction fragment length polymorphism (RFLP) patterns generated with *Hpy *99 I. Direct DNA sequencing was used to confirm RFLP results and for detecting mutations in *embC-embA *IGR.

**Results:**

Nearly same number of EMB-resistant *M. tuberculosis *strains were resistant to EMB alone and EMB together with additional resistance to rifampicin and isoniazid (9 of 50, 18% and 11 of 50, 22%, respectively). All the 25 pansusceptible strains contained wild-type sequences at *iniA501 *and in *embC-embA *IGR. The analysis of 50 EMB-resistant *M. tuberculosis *isolates showed that only one strain contained a mutated *iniA501 *while no mutation was detected in *embC-embA *IGR in any of the isolate.

**Conclusion:**

Analysis of *iniA501 *and *embC-embA *IGR in epidemiologically unrelated EMB-resistant *M. tuberculosis *isolates in Kuwait indicate that mutations at these locations occur very infrequently and their inclusion for the development of a comprehensive molecular screen will make only minor contribution towards rapid EMB resistance detection.

## Background

The tuberculosis (TB) epidemic continues unabated. Despite intense efforts made over the past two decades, the morbidity and mortality associated with TB remain high, with 8 million active disease cases and 2 million deaths occurring worldwide every year [1,2]. Two factors, co-infection with human immunodeficiency virus (HIV) and increasing incidence of infections with drug-resistant strains of *Mycobacterium tuberculosis *are steadily worsening the problem of TB [3,4]. The latest World Health Organization (WHO)-sponsored study showed that drug-resistant TB among new cases is prevalent in 74 of 77 (96%) settings or countries with resistance to at least one anti-TB drug varying from 0% in some rich, developed countries to >30% in several developing countries [4]. Infections with drug-resistant, particularly multidrug-resistant (MDR) (resistant to at least rifampicin and isoniazid) strains of *M. tuberculosis *(MDR-TB) are associated with higher mortality [4,5]. Molecular methods for rapidly identifying drug-resistant strains of *M. tuberculosis *are urgently needed to avoid inadequate treatment since phenotypic drug susceptibility testing by radiometric method requires 4–14 days after the primary culture has been isolated [6]. A simple and rapid line probe assay has been developed recently for detection of resistance of *M. tuberculosis *to rifampicin and isoniazid (MDR-TB) in cultured isolates and clinical samples [7]

Ethambutol (EMB), an arabinose analogue, is a bactericidal, first-line drug for the treatment of TB. The EMB is often used, along with isoniazid, rifampicin and pyrazinamide, as an alternative to streptomycin, in the four-drug regimens advocated by World Health Organization under the directly observed chemotherapy short-course. Global data on drug resistance patterns have shown that resistance of *M. tuberculosis *to EMB in newly diagnosed as well as previously treated cases is much less compared to streptomycin [4,8]. Similar pattern is also noted in the rate of resistance of *M. tuberculosis *isolates to EMB (1.98%) and streptomycin (4.31%) in Kuwait [9]. Since resistance of *M. tuberculosis *to EMB is also generally associated with resistance to other anti-TB drugs [8,10], early detection of resistance will not only abolish drug-associated adverse reactions, particularly optic neuritis, it will also suggest modifications in therapy regimens.

The mechanism of action and the molecular genetic basis of resistance to EMB are complex and are not completely defined. The enzymes participating in synthesis and polymerization of cell wall arabinan are implicated as the main target for EMB. These include three homologous and membrane associated arabinosyltransferases encoded by three contiguous genes, *embC-embA-embB*, isoniazid-inducible genes particularly *iniA*, acyl carrier proteins and regulatory proteins modulating their expression [11-13]. Mutations in *embB *particularly involving codon 306 (*embB306*) and less frequently, codons 406 (*embB406*) and 497 (*embB497*) have been identified as most common genetic alterations conferring resistance to EMB in clinical *M. tuberculosis *isolates [11-14]. The frequency of these mutations in EMB-resistant strains in some studies has been reported to be ~70% [14-17]. However, in epidemiologically unrelated strains, these mutations accounted for EMB resistance in only around 30% EMB-resistant isolates [13,18]. Only one study has so far detected mutations in other *emb *(*embA *and *embC*) or other genes conferring resistance to EMB in epidemiologically unrelated EMB-resistant strains [13]. The data showed that, mutations in *embC-embA *intergenic region (IGR) (four nucleotide positions), *embA *(several codons) and *iniA *(mainly codon 501, *iniA501*) occurred in ~30% EMB-resistant strains while mutations in eight other genes were either absent or occurred infrequently [13]. However, the role of mutations in these loci in conferring resistance to EMB remain unclear as these mutations, particularly in *embC-embA *IGR, were found in isolates with other well-characterized EMB resistance conferring mutations [13]. The TCG to TGG mutation at *iniA501 *most likely confers resistance of *M. tuberculosis *to EMB as it alters the structural property of the encoded protein [13]. However, the resistance conferring mechanism explaining the role of mutations in *embC-embA *IGR is not well characterized and the paucity of information on the frequency of mutations in *embC-embA *IGR in EMB-resistant *M. tuberculosis *strains from different regions of the world makes it difficult to ascertain their exact role in conferring resistance to EMB. The present study was carried out to detect the frequency of mutations at *iniA501 *and in *embC-embA *IGR in 50 consecutive and epidemiologically unrelated EMB-resistant *M. tuberculosis *strains that were analyzed previously for *embB306*, *embB406 *and *embB497 *mutations [18] in an effort to develop a more comprehensive molecular screen for EMB-resistance detection. The EMB-susceptible isolates were also analyzed simultaneously to ensure that such mutations are not present in EMB-susceptible strains.

## Materials and methods

### EMB-resistant and -susceptible *M. tuberculosis *strains

A single TB control unit and the Kuwait National Tuberculosis Reference Laboratory (KNTRL) under the Ministry of Health are responsible for the diagnosis and treatment of all TB patients in Kuwait. Appropriate specimens from suspected TB patients are sent to KNTRL for culture. All active TB cases are diagnosed by culture and susceptibility testing to first line anti-TB drugs is performed on all the isolates identified as *M. tuberculosis *[19]. All the 50 EMB-resistant *M. tuberculosis *strains (KE1 to KE50) isolated throughout Kuwait from 50 different TB patients during 2000 to 2003 were included in this study. The clinical background and demographic data of patients yielding EMB-resistant *M. tuberculosis *strains are shown in Table 1. Repeat isolates were recovered from eight TB patients within one to two months of isolation of the first isolate. A total of 25 *M. tuberculosis *strains susceptible to all first-line drugs (pansusceptible strains) were also included. The isolation and identification of *M. tuberculosis *strains from clinical specimens was performed using the mycobacterial growth indicator tube (MGIT) 960 system as described previously [20]. The drug susceptibility testing of *M. tuberculosis *isolates was performed using the BACTEC 460 TB system as reported earlier [6,21]. The isolates were considered EMB resistant when bacterial growth occurred at a concentration of 2.5 μg EMB per ml. Resistance of all the isolates to isoniazid (0.1 μg/ml), rifampicin (2 μg/ml) and streptomycin (2 μg/ml) was also determined.

**Table 1:** Clinical background, resistance patterns and demographic information of patients yielding EMB-resistant *M. tuberculosis *isolates in Kuwait

Isolate no.	Year of isolation	Clinical specimen^a^	Resistance pattern^b^	Patient demographics			
				
				Nationality	Region^c^	Age (years)	Sex
KE1	2000	Sputum	E, H, R	Indian	SA	43	Male

KE2	2000	Sputum	E, H, R	Indian	SA	45	Male

KE3	2000	Sputum	E, H	Egyptian	ME	47	Female

KE4	2000	Sputum	E, H, S	Indian	SA	27	Male

KE5	2000	Sputum	E, H	Indian	SA	42	Male

KE6	2000	Pus	E, H	Sri Lankan	SA	27	Male

KE7	2000	Abscess	E, H	Pakistani	ME	55	Male

KE8	2000	Sputum	E, H, S	Kuwaiti	ME	45	Male

KE9	2000	Pus	E, H, S	Indian	SA	45	Female

KE10	2000	Sputum	E, H	Bangladeshi	SA	40	Female

KE11	2001	Sputum	E, H	Filipino	SEA	35	Female

KE12	2001	Sputum	E, H	Indian	SA	22	Male

KE13	2001	Sputum	E, H, R	Indian	SA	47	Male

KE14	2001	Sputum	E, H, R, S	Indian	SA	27	Female

KE15	2001	Sputum	E, H, R, S	Indian	SA	32	Male

KE16	2001	FNA	E, H	Indian	SA	27	Female

KE17	2001	Sputum	E, H	Indian	SA	30	Male

KE18	2001	LN	E, H	Filipino	SEA	31	Male

KE19	2001	Sputum	E, H	Indian	SA	45	Male

KE20	2001	Sputum	E, H	Egyptian	ME	49	Male

KE21	2001	Sputum	E, H, S	Indian	SA	25	Male

KE22	2001	Sputum	E, H, R, S	Egyptian	ME	27	Female

KE23	2002	Sputum	E	Kuwaiti	ME	65	Male

KE24	2002	Sputum	E, H	Egyptian	ME	45	Male

KE25	2002	Pus	E, H	Kuwaiti	ME	34	Male

KE26	2002	FNA	E, S	Indian	SA	21	Male

KE27	2002	Pus	E	Indian	SA	30	Female

KE28	2002	BAL	E, H	Bangladeshi	SA	32	Male

KE29	2002	GS	E, R	Syrian	ME	57	Female

KE30	2002	Sputum	E, H, R, S	Nepalese	SA	23	Female

KE31	2002	FNA	E	Indian	SA	31	Male

KE32	2002	Sputum	E	Kuwaiti	ME	25	Female

KE33	2002	Sputum	E, H	Pakistani	ME	38	Male

KE34	2002	Sputum	E, H	Filipino	SEA	27	Female

KE35	2002	Sputum	E	Indian	SA	29	Male

KE36	2003	LN	E	Yemani	ME	31	Male

KE37	2003	Pus	E	Kuwaiti	ME	23	Male

KE38	2003	FNA	E, H, S	Pakistani	ME	23	Male

KE39	2003	Sputum	E	Indian	SA	26	Male

KE40	2003	FNA	E	Indian	SA	30	Male

KE41	2003	Sputum	E, H	Kuwaiti	ME	18	Female

KE42	2003	LN	E, H	Indian	SA	25	Female

KE43	2003	FNA	E, H	Kuwaiti	ME	37	Male

KE44	2003	Sputum	E, H	Pakistani	ME	54	Male

KE45	2003	PF	E, H	Indian	SA	45	Male

KE46	2003	Sputum	E, H, R, S	Indian	SA	47	Male

KE47	2003	Pus	E, H, R, S	Iranian	ME	35	Male

KE48	2003	Sputum	E, H, R, S	Egyptian	ME	24	Male

KE49	2003	FNA	E, H, R, S	Egyptian	ME	65	Female

KE50	2003	PF	E, H	Indian	SA	46	Male

^a^FNA, fine needle aspirate; LN, lymph node; BAL, bronchoalveolar lavage; GS, gastric secretion; PF, pleural fluid

^b^E, ethambutol; H, isoniazid; R, rifampicin; S, streptomycin

^c^SA, South Asia; ME, Middle East; SEA, Southeast Asia

### Sample preparation for PCR

The *M. tuberculosis *reference strain H_37_Rv was used as a control in PCR-RFLP and DNA sequencing. The reference strain and clinical EMB-resistant and pansusceptible *M. tuberculosis *isolates were obtained as heat killed BACTEC liquid cultures. One ml of BACTEC culture was heated with 40 mg Chelex-100 (Sigma) at 95°C for 20 min followed by centrifugation at 12,000 × g for 15 min and the supernatant obtained was used as the source of genomic DNA [22] for the amplification of various gene regions.

### Primer design and PCR amplification

Two primer pairs were synthesized. One primer pair (INIAF and INIAR) was used for the amplification of the *iniA *gene (Genbank accession no. Z95324) region around codon 501. Another primer pair (IGRF and IGRR) was synthesized for the amplification of *embC-embA *IGR (Genbank accession no. AL123456) including nucleotide positions that are mutated in EMB-resistant strains [13]. The DNA sequences of the primers, their positions on the target genes and the expected size of the PCR products are shown in Table 2.

**Table 2:** Primer sequences and their locations in target genes used for PCR amplification of *iniA501 *and *embC-embA *IGR

Primer pair	Primer name	Primer sequence	Target gene^a^	Direction^b^/position	Target region	Amplicon size (bp)	Mutations detected by
1	INIAF	5'-CGCTGGGCCGGATGGAATCGAA-3'	*iniA*	F, 1403–1424	Codon 501	241	*Hpy *99 I RFLP
	INIAR	5'-ACGAAGCGCCGCACATTGGCCTT-3'		R, 1643–1621			

2	IGRF	5'-GTTACAGCGGTTGACGCCTTACTA-3'	IGR^c^	F, 29061–29084	-11 to -43^d^	261	Sequencing
	IGRR	5'-CGATTCCCGAGACGACGGCTGCTA-3'		R, 29321–29298			

^a^The accession numbers for target genes are *iniA*, Z95324; *embC-embA *IGR, AL123456

^b^F, forward; R, reverse

^c^IGR, he *embC-embA *intergenic region

^d^-11 to -43 corresponds to -11, -12, -16 and -43 positions (relative to the start codon of *embA*) in *embC-embA *IGR

The PCR amplification of the target DNA was carried out in a final volume of 50 μl and contained 5 μl of 10× Perkin-Elmer PCR buffer, 10 pmol of the appropriate forward (F) and reverse (R) primers (INIAF and INIAR or IGRF and IGRR, Table 2), 2 μl of template DNA, 0.1 mM dNTPs, 2 units of AmpliTaq DNA polymerase (Perkin-Elmer) and sterile distilled water. The cycling parameters were same as described previously [22]. Following amplification, a portion of the product (10 μl) was run on 2% agarose gels [22] to confirm the amplification of a DNA fragment of expected size. The remaining PCR product was purified by using PCR purification columns (Qiagen) that were used as instructed by the manufacturer. The purified amplicons were used for further studies.

### Detection of mutations at *iniA501 *by PCR-RFLP

The mutations at *iniA501 *were detected by restriction digestion of amplicons obtained with primers INIAF and INIAR with *Hpy *99I as described previously [22]. Briefly, the reaction mixture in a final volume of 25 μl contained 5 μl of the purified amplicon, 10.25 μl of sterile water, 6.25 μl of NE Buffer 4, 2.5 μl of 10× BSA and 1 μl (2 units) of *Hpy99 *I (New England Biolabs). After incubation at 37°C for 1 h, the digested products were separated on 2.5% agarose gels. The amplicon from *M. tuberculosis *H_37_Rv as well as *M. tuberculosis *strains containing TCG at *iniA501 *(*iniA501TCG*) yield two fragments of 104 bp and 137 bp while the amplicon from isolates containing TGG at *iniA501 *(*iniA501TGG*) yield the original undigested fragment of 241 bp [22]. The results of RFLP for some of the isolates were confirmed by DNA sequencing. The DNA sequencing was performed by using the cycle DNA sequencing kit (DTCS CEQ2000, Beckman Coulter) as described in detail previously [22]. Briefly, the reaction mixtures in a final volume of 20 μl contained, 8.0 μl of purified amplicon, 1.0 μl (3.2 pmol) of primer INIAF or INIAR (Table 2) or an internal primer (INIAS, 5'-CGCTGGGCCGGATGGAATCGAA-3'), 8.0 μl of the pre-mix reaction components supplied in the kit and 3.0 μl sterile water. The cycling parameters were same as described previously [22]. Reactions products were precipitated and loaded on the DNA sequencer as directed by the manufacturer (Beckman Coulter Model CEQ8000).

### Detection of mutations in *embC-embA *IGR by DNA sequencing

The mutations at -11, -12, -16 and -43 positions (relative to the translational start point of *embA*) in *embC-embA *IGR [13] were detected by direct DNA sequencing of purified amplicons, obtained by using IGRF and IGRR primers. The DNA sequencing was carried out, as described above except that primer IGRF or IGRR (Table 2) or an internal primer (IGRS, 5'-CCGCTGATCTGAACCTAGGAAC-3') was used as sequencing primer.

### Molecular fingerprinting of EMB-resistant *M. tuberculosis *isolates

The molecular fingerprinting of EMB-resistant *M. tuberculosis *isolates was performed by genetic group analysis [23] based on polymorphisms at *katG *codon 463 (*katG463*) and *gyrA *codon 95 (*gyrA95*) and by touchdown double-repetitive-element (DRE)-PCR. The presence of Arg463/Leu463 at *katG463 *and Ser95/Thr95 at *gyrA95 *were determined by PCR-RFLP as described previously [24,25]. Briefly, the amplification of *katG463 *and *gyrA95 *DNA regions was carried out as described above except that primers KatG1F (5'-CCCGAGGAATTGGCCGACGAGTTC-3') + KatG1R (5'-GGTGCGAATGACCTTGCGCAGATC-3') [24] and GYRA95F (5'-CGCAGCTACATCGACTATGCGATG-3') + GYRA95R (5'-GGGCTTCGGTGTACCTCATCGCC-3') [25], respectively, were used. The purified *katG463 *region amplicons were digested with restriction enzyme *Nci *I to obtain RFLP patterns. The reaction mixture in a final volume of 25 μl contained 2.5 μl of BRL Buffer 8 (Gibco-BRL), 5 μl of amplicon, 2 μl (10 units) of *Nci *I (Gibco-BRL), and 15.5 μl of sterile water. After incubation at 37°C for 4 h, the digested products were separated on 2.5% agarose gels. The amplicon from *M. tuberculosis *H_37_Rv as well as *M. tuberculosis *strains containing Arg at *katG463 *(*katG463CGG*) yield two fragments of 187 bp and 173 bp while the amplified fragment from isolates containing Leu at *katG463 *(*katG463CTG*) yield the original undigested fragment of 360 bp [24]. The purified *gyrA95 *region amplicons were digested with restriction enzyme *Ale *I to obtain RFLP patterns. The reaction mixture in a final volume of 25 μl contained, 2.5 μl of NE Buffer 4, 1.25 μl of 10× BSA (New England Biolabs), 6.0 μl of the amplified DNA, 1 μl (5 units) of *Ale *I and 14.25 μl of sterile water. After incubation at 37°C for 3 h, the digested products were separated on 2.5% agarose gels. The amplicon from *M. tuberculosis *H_37_Rv as well as *M. tuberculosis *strains containing Ser at *gyrA95 *(*gyrA95AGC*) yield the original undigested fragment of 322 bp while the amplified fragment from isolates containing Thr at *gyrA95 *(*gyrA95ACC*) yield two fragments of 212 bp and 110 bp [25].

The typing of EMB-resistant *M. tuberculosis *isolates was performed by touchdown DRE-PCR as described previously [26]. Briefly, the reaction mixtures in a final volume of 50 μl contained 1× Perkin-Elmer PCR buffer II, 2.5 mM MgCl_2_, 10 pmol each of the four primers (IS6110-5'R, 5'-GGCTGAGGTCTCAGATCAGAG-3'; IS6110-3'F, 5'-ACCCCATCCTTTCCAAGAACT-3'; PGRS-5'R, 5'-TCCCCGCCGTTGCCGTACAG-3' and PGRS-3'F, 5'-CTTGGGAAACCCGGCCAGCTG-3') [26], 2 μl of template DNA, 0.2 mM dNTPs and 2 units of AmpliTaq DNA polymerase. The cycling parameters were same as described previously [26]. The amplified products (20 to 25 μl) were resolved on 2% agarose gels. The *M. tuberculosis *isolates belonging to different genetic groups and/or yielding unique patterns of DNA amplified fragments in DRE-PCR were considered as genotypically distinct strains.

### Nucleotide sequence accession numbers

The DNA sequencing data for the mutant strain reported in this study have been deposited in EMBL under the accession number AJ973188.

## Results and Discussion

A total of 50 EMB-resistant strains of *M. tuberculosis *were isolated from 50 different TB patients throughout Kuwait during the year 2000 to 2003 and all the isolates were included for analysis. Twenty of these isolates were from patients of Middle-Eastern origin (including seven Kuwaiti nationals), 27 from patients of South-Asian origin and three from patients of Southeast Asian countries (Table 1). All the isolates were recovered from HIV-negative adult TB patients (18 to 65 years) and included 15 female and 35 male patients. The clinical specimens yielding these strains included sputum (n = 29), pus (n = 6), fine needle aspirate (n = 7), lymph node (n = 3), pleural fluid (n = 2), abscess (n = 1), bronchoalveolar lavage (BAL) (n = 1), and gastric secretion (n = 1) (Table 1). In addition, 25 pansusceptible clinical *M. tuberculosis *isolates were also included in the study. Nine, 12 and four of these isolates were from patients of Middle-Eastern (including three Kuwaiti nationals), South Asian, and Southeast Asian origin, respectively. The clinical specimens yielding pansusceptible strains included sputum samples (n = 15), pus (n = 6), fine needle aspirate (n = 3), and tissue biopsy (n = 1). The data are consistent with previously reported observations that majority (~80%) of *M. tuberculosis *infections in Kuwait occur in foreign-born expatriate workers mostly within the first few years of their migration even though all expatriates entering Kuwait are screened for TB (chest radiograph) as well as for HIV infection [9,27].

Only nine of 50 (18%) EMB-resistant strains were monoresistant to EMB while the remaining (41 of 50, 82%) isolates were additionally resistant to at least one more first-line drug. Most (39 of 41) of the latter isolates were resistant to isoniazid with or without additional resistance to other drugs. Twenty-four (48%) *M. tuberculosis *strains were resistant to two drugs while eight (16%) isolates were resistant to three drugs (including three MDR-TB strains). Only eight (16%) EMB-resistant strains were also resistant to the other three first-line drugs. All the isolates were resistant to EMB on first isolation indicating that the patients were already infected with EMB-resistant strains. Furthermore, nearly same number (9 of 50, 18% and 11 of 50, 22%) of EMB-resistant *M. tuberculosis *strains were resistant to EMB alone or EMB together with additional resistance to rifampicin and isoniazid (MDR-TB strains). Worldwide, monoresistance to EMB is rare and majority of EMB-resistant strains particularly those originating from TB endemic countries are MDR-TB strains [8,17,28].

The PCR amplification of *iniA501 *DNA region from 25 pansusceptible and 50 EMB-resistant *M. tuberculosis *strains yielded an amplicon of 241 bp, as expected. The *Hpy *99 I digestion patterns of purified amplicons showed that all the 25 pansusceptible and 49 EMB-resistant strains contained TCG at *iniA501 *(*iniA501TCG*) while only one EMB-resistant strain contained a mutated *iniA501*. The results of PCR-RFLP for three pansusceptible strains containing *iniA501TCG*, three EMB-resistant strains also containing *iniA501TCG *and one EMB-resistant strain containing a mutated *iniA501 *were confirmed by DNA sequencing. The solitary isolate with a mutation at *iniA501 *contained *iniA501TGG *(Table 3). Likewise, the PCR amplification of DNA region encompassing *embC-embA *IGR from 25 pansusceptible and 50 EMB-resistant *M. tuberculosis *strains also yielded an amplicon of 261 bp, as expected. Direct DNA sequencing of the purified amplicons showed that all the 25 pansusceptible and 50 EMB-resistant strains contained wild-type sequences including nucleotide positions -11, -12, -16 and -43 [12] relative to the translational start point for *embA *gene (Table 3). The repeat isolates recovered from eight TB patients yielded the same resistance pattern and the same results for *iniA501 *and *embC-embA *IGR analyses as the parent isolate (data not shown).

**Table 3:** Occurrence of mutations at *iniA501 *and *embC-embA *IGR in 25 pansusceptible and 50 EMB-resistant clinical *M. tuberculosis *strains isolated in Kuwait

Susceptibility to ethambutol	No. of isolates	No. of isolates with *iniA501 *as		No. of isolates with wild-type nucleotides at -11, -12, -16 and -43 in *embC-embA *IGR
			
		Wild-type (TCG)	Mutant (TGG)	
Susceptible	25	25	0	25

Resistant	50	49	1	50

Consistent with previously reported data showing that majority of rifampicin-resistant and isoniazid-resistant *M. tuberculosis *infections in Kuwait occur in foreign-born TB patients as a result of reactivation of previously acquired infection [21,29,30], genetic group analysis and fingerprinting patterns obtained in DRE-PCR showed that majority of EMB-resistant *M. tuberculosis *strains were also unique strains (data not shown).

Majority (~70%) of EMB-resistant *M. tuberculosis *strains from several geographical locations around the world have been shown to contain mutations within *embB *gene particularly at *embB306*, *embB406 *and *embB497 *[14-17]. However, in most of these studies, all or consecutive EMB-susceptible and EMB-resistant *M. tuberculosis *strains from a single location and/or isolated over a specified period of time were not included and the proportion of MDR-TB strains or strains resistant to several anti-TB drugs was unusually high [15-17]. When the 50 phenotypically documented EMB-resistant *M. tuberculosis *strains used in this study were tested for genotypic resistance detection targeting mutations at *embB306*, *embB406 *and *embB497*, only 38% (19 of 50) isolates contained a resistance conferring mutation [17]. These findings suggested that mutations at other codon positions within *embB *gene or in other loci are responsible for conferring resistance to EMB in majority of EMB-resistant *M. tuberculosis *strains in Kuwait and their identification will be crucial for the development of a molecular screen for rapid detection of majority of EMB-resistant strains.

The results presented in this study showed that mutations at *iniA501 *and in *embC-embA *IGR in EMB-resistant *M. tuberculosis *strains in Kuwait also occur rather infrequently (1 of 50, 2%). Thus, their inclusion in the molecular screen will have only minor contribution towards detection of EMB resistance in Kuwait. Only one previous study has detected the occurrence of mutations at *iniA501 *and in *embC-embA *IGR in EMB-resistant *M. tuberculosis *strains [13]. Although the authors reported that mutations at *iniA501 *and in *embC-embA *IGR occurred in nearly 20% (15 of 75) of EMB-resistant strains, majority of isolates with mutations at *iniA501 *and all the isolates with a mutation in *embC-embA *IGR also contained other well characterized EMB resistance-conferring mutations, particularly at *embB306*, *embB406 *and *embB497 *[13]. Previous studies have shown that drug-resistant *M. tuberculosis *strains isolated from TB patients with limited previous exposure to first-line anti-TB drugs usually contain single point mutations in resistance conferring genes while strains from previously treated patients usually contain multiple mutations in target genes [15,18,20,31-33]. The susceptibility of EMB-resistant strains to other first-line drugs and the HIV status of the TB patients in the study that analyzed mutations at *iniA501 *and in *embC-embA *IGR was not indicated [13]. However, the presence of mutations in two or more genes involved in EMB resistance in 25% (19 of 75) EMB-resistant strains and the geographical origin of the tested isolates indicates that the isolates were either MDR-TB strains or strains additionally resistant to multiple first-line drugs [13]. These observations are also consistent with the role of mutations at *iniA501 *and in *embC-embA *IGR in conferring resistance to EMB.

The TCG to TGG mutation at *iniA501 *most likely confers resistance of *M. tuberculosis *to EMB by altering the structural property of the encoded protein [13]. However, the resistance conferring mechanism explaining the role of mutations in *embC-embA *IGR in EMB resistance is not well characterized. The suggestion that C to T mutations at -12 and -16 positions lead to creation of TATA box-like sequences [13] seems less likely since these elements are found in promoter elements while the hot-spot DNA segment is immediately upstream of the translational start point for EmbA and putatively involves ribosomal binding site. Furthermore, other characteristic features of promoter elements (such as -35 element sequences) are also absent in the surrounding DNA region. An alternative (titration) mechanism to explain the role of mutations in *embC-embA *IGR is summarized below and most likely involves premature termination of some transcripts originating from *embCAB *promoter before *embA *and *embB *genes are transcribed. The *embC-embA *IGR of 85 nucleotides is rather long for a polycistronic bacterial mRNA. A closer look at *embC-embA *IGR and N-terminal region of *embA *indicates that it contains an I-shaped transcriptional terminator typically found in mycobacteria including *M. tuberculosis *[34]. The RNA transcript involving -19 to +23 nucleotides (relative to translational start site of *embA*) can form a hairpin structure that is stabilized by nine G.C pairs including the two 'C' residues (at -12 and -16 positions) that are mostly mutated in some EMB-resistant strains [13]. Thus, mutations of -12C or -16C residues will decrease the stability of the hair-pin structure resulting in decreased termination of transcripts and consequently, increased expression of *embA *and *embB *encoded gene products. These assumptions are further supported by occurrence of nearly all the mutations in *embC-embA *IGR in isolates with a mutated *embB *gene. The above also explains the absence of these mutations in EMB-resistant *M. tuberculosis *isolates in Kuwait as such mutations are more likely to occur in TB patients with previous history of prolonged therapy with anti-TB drugs [15,18,20,31-33].

## Conclusion

In conclusion, the results presented in this study showed that mutations at *iniA501 *and in *embC-embA *IGR in EMB-resistant *M. tuberculosis *strains isolated from TB patients in Kuwait occur infrequently. Consequently, inclusion of mutation detection at these loci in the molecular screen for detecting majority of EMB-resistant *M. tuberculosis *strains will contribute minimally towards this goal in Kuwait as well as, perhaps, in other countries. Further studies from other geographical regions are clearly warranted to validate our observations.

## Competing interests

The authors declare that they have no competing interests.

## Authors' contributions

SA and EM participated in the design of the study, analyzed the data and drafted the manuscript. They also arranged financial support for the study. A-AJ carried out the experimental work. All authors have read and approved the final manuscript.

## References

[B1] Harries AD, Dye C (2006). Tuberculosis. Ann Trop Med Parasitol.

[B2] World Health Organization Global tuberculosis control: surveillance, planning, financing. WHO report 2007. Geneva, Switzerland, World Health Organization. WHO/HTM/TB/2007.

[B3] Wells CD, Peter Cegielski J, Nelson LJ, Laserson KF, Holtz TH, Finlay A, Castro KG, Weyer K (2007). HIV infection and multidrug-resistant tuberculosis-the perfect storm. J Infect Dis.

[B4] Aziz MA, Wright A, Laszlo A, De Muynck A, Portaels F, Van Deun A, Wells C, Nunn P, Blanc L, Raviglione M, for the WHO/International Union Against Tuberculosis and Lung Disease global project on anti-tuberculosis drug resistance surveillance (2006). Epidemiology of antituberculosis drug resistance (the Global Project on Anti-tuberculosis Drug Resistance Surveillance): an updated analysis. Lancet.

[B5] Zignol M, Hosseini MS, Wright A, Lambregts-van Weezenbeek C, Nunn P, Watt CJ, Williams BG, Dye C (2006). Global incidence of multidrug-resistant tuberculosis. J Infect Dis.

[B6] Siddiqi SH, Hawkins J, Laszlo A (1985). Interlaboratory drug susceptibility testing of *Mycobacterium tuberculosis *by radiometric procedure and two conventional methods. J Clin Microbiol.

[B7] Lacoma A, Garcia-Sierra N, Prat C, Ruiz-Manzano J, Haba L, Rosés S, Maldonado J, Domínguez J (2008). GenoType MTBDRplus assay for molecular detection of rifampin and isoniazid resistance in *Mycobacterium tuberculosis *strains and clinical samples. J Clin Microbiol.

[B8] Espinal MA, Laszlo A, Simonsen L, Boulahbal F, Kim SJ, Reniero A, Hoffner S, Rieder HL, Binkin N, Dye C, Williams R, Raviglione MC, for the World Health Organization-International Union Against Tuberculosis and Lung Disease working group on anti-tuberculosis drug resistance surveillance. (2001). Global trends in resistance to antituberculosis drugs. N Engl J Med.

[B9] Mokaddas E, Ahmad S, Samir I (2008). Secular trends in susceptibility patterns of *Mycobacterium tuberculosis *isolates in Kuwait, 1996–2005. Int J Tuberc Lung Dis.

[B10] Cavusoglu C, Hilmioglu S, Guneri S, Bilgic A (2006). Characterization of *rpoB *mutations in rifampin-resistant *Mycobacterium tuberculosis *isolates from Turkey by DNA sequencing and line probe assay. J Clin Microbiol.

[B11] Alcaide F, Pfyffer GE, Telenti A (1997). Role of *embB *in natural and acquired resistance to ethambutol in mycobacteria. Antimicrob Agents Chemother.

[B12] Telenti A, Phillipp W, Sreevatsan S, Bernasconi C, Stockbauer KE, Wieles B, Musser JM, Jacobs WR (1997). The *emb *operon, a unique gene cluster of *Mycobacterium tuberculosis *involved in resistance to ethambutol. Nat Med.

[B13] Ramaswamy S, Amin AG, Koksel S, Stager CE, Dou S-J, El Sahly H, Moghazeh SL, Kreiswirth BN, Musser JM (2000). Molecular genetic analysis of nucleotide polymorphisms associated with ethambutol resistance in human isolates of *Mycobacterium tuberculosis*. Antimicrob Agents Chemother.

[B14] Sreevatsan S, Stockbauer KE, Pan X, Kreiswirth BN, Moghazeh SL, Jacobs WR, Telenti A, Musser JM (1997). Ethambutol resistance in *Mycobacterium tuberculosis *: critical role of *embB *mutations. Antimicrob Agents Chemother.

[B15] Ramaswamy SV, Dou S-J, Rendon A, Yang Z, Cave MD, Graviss EA (2004). Genotypic analysis of multidrug-resistant isolates from Monterrey, Mexico. J Med Microbiol.

[B16] Parsons LM, Salfinger M, Clobridge A, Dormandy J, Mirabello L, Polletta VL, Sanic A, Sinyavskiy O, Larsen SC, Driscoll J, Zickas G, Taber HW (2005). Phenotypic and molecular characterization of *Mycobacterium tuberculosis *isolates resistant to both isoniazid and ethambutol. Antimicrob Agents Chemother.

[B17] Pinke C, Rusch-Gerdes S, Nieman S (2006). Significance of mutations in *embB *codon 306 for prediction of ethambutol resistance in clinical *Mycobacterium tuberculosis *isolates. Antimicrob Agents Chemother.

[B18] Ahmad S, Jaber A-A, Mokaddas E (2007). Frequency of *embB *codon 306 mutations in ethambutol-susceptible and -resistant clinical *Mycobacterium tuberculosis *isolates in Kuwait. Tuberculosis.

[B19] Mokaddas E, Ahmad S (2007). Development and evaluation of a multiplex PCR for rapid detection and differentiation of *Mycobacterium tuberculosis *complex members from non-tuberculous mycobacteria. Jpn J Infect Dis.

[B20] Ahmad S, Mokaddas E (2005). The occurrence of rare *rpoB *mutations in rifampicin-resistant *Mycobacterium tuberculosis *isolates from Kuwait. Int J Antimicrob Agents.

[B21] Ahmad S, Mokaddas E, Fares E (2002). Characterization of *rpoB *mutations in rifampin-resistant clinical *Mycobacterium tuberculosis *isolates from Kuwait and Dubai. Diagn Microbiol Infect Dis.

[B22] Ahmad S, Mokaddas E, Jaber A-A (2004). Rapid detection of ethambutol-resistant *Mycobacterium tuberculosis *strains by PCR-RFLP targeting *embB *codons 306 and 497 and *iniA *codon 501 mutations. Mol Cell Probes.

[B23] Sreevatsan S, Pan X, Stockbauer KE, Connell ND, Kreiswirth BN, Whittam TS, Musser JM (1997). Restricted structural gene polymorphism in the *Mycobacterium tuberculosis *complex indicates evolutionarily recent global dissemination. Proc Natl Acad Sci USA.

[B24] Ahmad S, Mokaddas E, Abal AT, Araj GF, Fares E, Mustafa AS (2001). Genetic polymorphism at codon 463 in the *katG *gene in isoniazid-sensitive and -resistant *Mycobacterium tuberculosis *isolates from the Middle East. Med Princ Pract.

[B25] Mokaddas E, Ahmad S, Abal AT, Al-Shami AS (2005). Molecular fingerprinting reveals familial transmission of rifampin-resistant tuberculosis in Kuwait. Ann Saudi Med.

[B26] Mokaddas E, Ahmad S, Abal AT (2002). Molecular fingerprinting of isoniazid-resistant *Mycobacterium tuberculosis *isolates from Chest Disease Hospital in Kuwait. Microbiol Immunol.

[B27] Mokaddas E, Ahmad S (2008). Species spectrum of nontuberculous mycobacteria isolated from clinical specimens in Kuwait. Curr Microbiol.

[B28] Hazbon MH, Bobadilla del Valle M, Guerrero MI, Varma-Basil M, Filliol I, Cavatore M, Colangeli R, Safi H, Billman-Jacobe H, Lavender C, Fyfe J, Garcia-Garcia L, Davidow A, Brimacombe M, Leon CI, Porras T, Bose M, Chaves F, Eisenach KD, Sifuentes-Osornio J, Ponce de Leon A, Cave MD, Alland D (2005). Role of *embB *codon 306 mutations in *Mycobacterium tuberculosis *revisited: a novel association with broad drug resistance and IS6110 clustering rather than ethambutol resistance. Antimicrob Agents Chemother.

[B29] Abal AT, Ahmad S, Mokaddas E (2002). Variations in the occurrence of the S315T mutation within the *katG *gene in isoniazid-resistant clinical *Mycobacterium tuberculosis *isolates from Kuwait. Microb Drug Resist.

[B30] Ahmad S, Mokaddas E (2004). Contribution of AGC to ACC and other mutations at codon 315 of the *katG *gene in isoniazid-resistant *Mycobacterium tuberculosis *isolates from the Middle East. Int J Antimicrob Agents.

[B31] Cardoso RF, Cooksey RC, Morlock GP, Barco P, Cecon L, Forestiero F, Leite CQF, Sato DN, Shikama MdeL, Mamizuka EM, Hirata RDC, Hirata MH (2004). Screening and characterization of mutations in isoniazid-resistant *Mycobacterium tuberculosis *isolates obtained in Brazil. Antimicrob Agents Chemother.

[B32] Siddiqi N, Shamim M, Hussain S, Choudhary RK, Ahmed N, Prachee, Banerjee S, Savithri GR, Alam M, Pathak N, Amin A, Hanief M, Katoch VM, Sharma SK, Hasnain SE (2002). Molecular characterization of multidrug-resistant isolates of *Mycobacterium tuberculosis *from patients in North India. Antimicrob Agents Chemother.

[B33] Caws M, Duy PM, Tho DQ, Lan NT, Hoa DV, Farrar J (2006). utations prevalent among rifampin- and isoniazid-resistant *Mycobacterium tuberculosis *Misolates from a hospital in Vietnam. J Clin Microbiol.

[B34] Mitra A, Angamuthu K, Nagaraja V (2008). Genome-wide analysis of the intrinsic terminators of transcription across the genus *Mycobacterium*. Tuberculosis.

